# Early changes in arterial structure and function following statin initiation: Quantification by magnetic resonance imaging

**DOI:** 10.1016/j.atherosclerosis.2007.09.001

**Published:** 2008-04

**Authors:** Justin M.S. Lee, Frank Wiesmann, Cheerag Shirodaria, Paul Leeson, Steffen E. Petersen, Jane M. Francis, Clare E. Jackson, Matthew D. Robson, Stefan Neubauer, Keith M. Channon, Robin P. Choudhury

**Affiliations:** Department of Cardiovascular Medicine, University of Oxford, United Kingdom

**Keywords:** Statin, Magnetic resonance imaging, Aorta, Carotid, Atherosclerosis

## Abstract

Effective LDL-cholesterol (LDL-C) reduction improves vascular function and can bring about regression of atherosclerosis. Alterations in endothelial function can occur rapidly, but changes in atherosclerosis are generally considered to occur more slowly. Vascular magnetic resonance imaging (MRI) is a powerful technique for accurate non-invasive assessment of central and peripheral arteries at multiple anatomical sites. We report the changes in atherosclerosis burden and arterial function in response to open label statin treatment, in 24 statin-naïve newly diagnosed stable coronary artery disease patients. Patients underwent MRI before, and 3 and 12 months after commencing treatment. Mean LDL-C fell by 37% to 70.8 mg/dL (*P* < 0.01). The plaque index (normalised vessel wall area) showed reductions in the aorta (2.3%, *P* < 0.05) and carotid (3.1%, *P* < 0.05) arteries at 3 months. Early reductions in atherosclerosis of aorta and carotid observed at 3 months were significantly correlated with later change at 12 months (*R*^2^ = 0.50, *P* < 0.001; *R*^2^ = 0.22, *P* < 0.05, respectively). Improvements in aortic distensibility and brachial endothelial function that were apparent after 3 months treatment were sustained at the 12-month time point.

## 1 Introduction

HMG CoA reductase inhibitors or ‘statins’ reduce cardiovascular events by approximately 25–30% in patients with stable atherosclerotic disease [1,2]. Previous studies of ultrasound carotid intima media thickness (CIMT) have suggested relatively slow regression of atherosclerosis after 1–2 years of high dose statin treatment [3,4]. More recently, intravascular ultrasound (IVUS) of the coronary arteries has demonstrated coronary plaque regression following intensive LDL-C reduction in over 300 patients treated with 40 mg rosuvastatin for 2 years [5]. Corti et al. [6] were among the first to use serial magnetic resonance imaging (MRI), to demonstrate reduction in carotid and aortic atherosclerosis in 18 patients in response to 12 months of statin treatment. Subsequently, the same group demonstrated that more effective lipid lowering, to LDL-C < 100 mg/dL, was associated with a larger decrease in plaque size at 12 months [7]. Similarly, Saam et al. [8] have recently identified statin treatment as an independent predictor of reduced annual rate of carotid atheroma progression, measured using MRI.

In the setting of acute coronary syndromes, early initiation of ‘intensive’ statin treatment can rapidly reduce cardiovascular risk within 4–16 weeks [9,10]. However, given the apparently slow changes in atheroma burden observed in previous studies [6], it has been hypothesised that the early clinical benefits of statins cannot reflect structural changes within arteries, but are due to ‘pleiotropic’ effects of statins such as improvement in endothelial function and reduction in inflammation and thrombosis [11]. Indeed, numerous studies have demonstrated rapid improvement in endothelial function in response to statin treatment [12–14]. However, there is also evidence that rapid changes in plaque size and composition might also be possible. Animal models of atherosclerosis indicate that potent correction of dyslipidaemia can result in prompt regression and favourable remodelling of plaques after only 9 weeks [15]. Furthermore, in humans, a recent study of treatment with intravenous apoAI-Milano induced modest but significant regression of coronary atherosclerosis after only 6 weeks [16].

We have previously shown that MRI can assess both central and peripheral vascular function, including measurements of arterial stiffness and endothelial function [17,18]. These parameters are of additional interest because they have been shown prospectively to predict cardiovascular risk [19,20]. By assessing both atherosclerosis and vascular function in the same patients we hoped to examine the extent to which changes in structural parameters might be anticipated by early functional changes. In this study, we used magnetic resonance imaging to evaluate, in vivo, the changes in structure and function in human aorta, carotid and brachial arteries at 0, 3 and 12 months in response to statin therapy. Notably, the mean post-treatment LDL-C achieved in this study reached the contemporary target of 70 mg/dL [21].

## 2 Methods

### 2.1 Study population

Newly diagnosed patients (*n* = 32) with coronary artery disease were recruited from the Cardiology Department of a single tertiary centre. Diagnosis was based on a history of typical symptoms of angina together with an exercise ECG that showed ischaemic-type ST segment changes or a stenosis of ≥50% in at least one coronary artery at angiography. No patients had taken statins prior to study enrolment. No other cholesterol modifying agents were permitted, but no further restrictions were placed on concomitant cardiovascular medications. Patients with acute coronary syndromes or contra-indication to MRI were excluded. MRI was performed at baseline, 3 and 12 months. At each time point, venous blood samples were obtained after a 12-h fast. Statin treatment was withheld until immediately after the first MRI scan, when a statin was started according to the preference of the managing clinician. Decisions regarding cholesterol treatment and management of cardiac risk factors were taken by the responsible clinicians. Statin dose titration, whilst allowed, was not protocol-driven. The study protocol was approved by the local Research Ethics Committee and all subjects gave informed written consent. Of the 32 subjects initially recruited, 8 did not complete all three study time points: 1 patient died from an out of hospital cardiac arrest, 5 were unwilling to return for follow-up scans due to claustrophobia and 2 were lost to follow-up. The 8 patients who did not complete the study were not significantly different from the 24 who did in terms of either their risk factors (age, cholesterol, diabetes, hypertension and smoking) or MRI measures of atheroma or vascular function at baseline. The data reported below refer to the 24 patients, who completed the study protocol.

### 2.2 Vascular MRI protocol

**Fig. 1:**
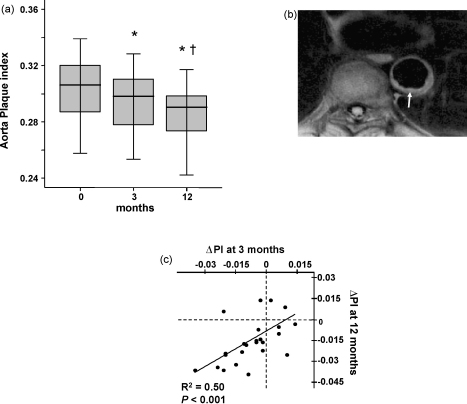
(a) Plaque index in the aorta is reduced in response to statin treatment. Significant reductions were observed between baseline and 3 months and between 3 and 12 months (* indicates *P* < 0.05 vs. baseline, † indicates *P* < 0.05 vs. 3 months). (b) Proton density weighted image of descending thoracic aorta showing a well-defined, eccentric, mildly lobulated plaque in the descending thoracic aorta (arrow). (c) Change in plaque index (*Δ*PI) at 3 months was significantly correlated to the change measured at 12 months.

**Fig. 2:**
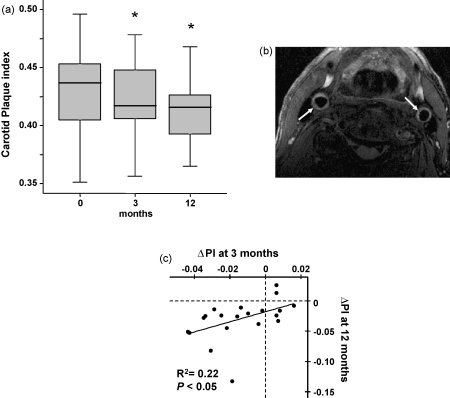
(a) Plaque index in the carotid arteries was reduced in response to statin treatment. Significant reductions were observed between baseline and 3 months but not between 3 and 12 months (* indicates *P* < 0.05 vs. baseline). (b) T2 weighted magnetic resonance image of the common carotid arteries (arrows) demonstrating bilateral non-obstructive common carotid plaques. (c) Change in plaque index (*Δ*PI) at 3 months was significantly correlated to the change measured at 12 months.

Imaging was performed on a 1.5 T magnetic resonance scanner (Siemens Sonata, Erlangen, Germany) as previously described [17,18]. In brief, aortic imaging was performed using a combination of a two-element array surface coil placed on the chest and spine coil array. For carotid artery imaging, a two-element array surface coil was used (Machnet BV, Eelde, Netherlands) and for brachial artery imaging a surface coil was attached at the right elbow. Brachial artery blood pressure was monitored using a blood pressure cuff on the left arm. For quantification of aortic wall area, ECG-gated double-inversion recovery (black-blood) fast spin echo images were acquired during breath-hold (Figs. 1b and 2b). Typical parameters were FOV 200 mm, TR 750 ms, TE 11 ms, in plane resolution 0.8 mm × 0.8 mm, slice thickness 5 mm. Using an oblique sagittal image of the aorta as a pilot, 11 serial axial images were acquired with 5 mm interslice gap to cover the entire descending thoracic aorta. The midpoint of the right pulmonary artery in cross section was used as the anatomical reference for the first slice in baseline and follow-up scans. For the carotid arteries axial ECG-gated T2 weighted, black blood images of the neck were acquired during free breathing (Fig. 2b). Sequence parameters: FOV 150 mm, TR 2 R–R intervals, TE 81 ms, resolution 0.5 mm × 0.5 mm in plane, slice thickness 3 mm, no interslice gap. Nine images of the common carotid artery were acquired using the common carotid bifurcation as the anatomical reference position for baseline and follow-up scans. Care was taken to place aortic and carotid image slices perpendicular to the long axis of the vessel on the pilot images in order to limit partial volume effects. For analysis of aorta and carotid plaque images all identifying data were removed apart from a code number so that observers were blinded to both patient identity and study time point. The external vessel boundary and vessel lumen were contoured manually by one of two experienced observers (J.L., C.S.) using CMRtools software (Imperial College, London, UK). Vessel wall area was calculated from the difference between these two contours, and then normalised to external vessel area to yield a plaque index (PI), as previously described [22,23]. Plaque index for each patient was then expressed as the mean of all aortic or carotid slices. In keeping with previous studies of carotid atherosclerosis, a mean value was obtained for left and right carotid arteries combined [3,4]. A subset of images from four randomly selected patients (>100 images) were analysed by both observers to assess inter-observer variability.

Steady state free precession (SSFP) acquisitions were used to determine aortic distensibility and brachial artery reactivity, as previously described [17,18]. Post processing of aortic and brachial images was performed using semi-automated edge detection methods developed with Matlab software (Mathworks Inc.) [24]. Maximum and minimum aortic cross-sectional areas over the cardiac cycle were measured, from which distensibility was calculated as the relative change in area divided by the pulse pressure. To assess brachial artery flow mediated dilatation (FMD), cross sectional images of the brachial artery were acquired at baseline and following release of a cuff inflated to 50 mmHg above systolic blood pressure on the forearm for 5 min. After 10 min, further brachial artery images were acquired following administration of 400 μg of sublingual glyceryl trinitrate to elicit maximal (endothelial-independent) dilatation. Maximum percentage change in cross sectional area was used to determine the response to each stimulus.

### 2.3 Serum and plasma assays

Cholesterol and lipoprotein assays were performed using a Cobas-Mira Analyser (ABX Diagnostics, Shefford, UK). Total cholesterol was assayed using the enzymatic CHOD-PAP method and triglycerides were assayed using the enzymatic GPO-PAP method. HDL-cholesterol was assayed using a homogenous second generation PEGME method (Roche Diagnostics, Burgess Hill, UK). Apolipoprotein AI (apoAI) and apolipoprotein B (apoB) were assayed using immunoturbidimetric methods, using reagents supplied by ABX Diagnostics. C-reactive protein was analysed using ELISA (MP Biomedicals, UK) according to the manufacturer's instructions.

### 2.4 Statistical analyses

Statistical analysis was performed using SPSS 12.0 (SPSS Inc., Chicago IL). The Kolmogorov–Smirnov test was used to assess whether data were normally distributed. Measurements at each time point were compared using repeated measures ANOVA for normally distributed data and Friedman analysis of variance by ranks for non-normally distributed data. Post-hoc analysis of paired time points was performed using a Bonferroni correction. Categorical data were analysed by the *χ*^2^ test. Data are presented as mean ± standard deviation or median and interquartile range as appropriate. Statistical significance was assigned at *P* < 0.05.

## 3 Results

### 3.1 Clinical and biochemical measures

Baseline characteristics of the 24 patients who completed all three study time points are shown in Table 1. The most common statin dose used was simvastatin 40 mg daily (63% patients); other statin prescriptions are detailed in Table 1. Mean baseline total cholesterol was 187.9 mg/dL (LDL-C 112.7 mg/dL), however 3 months after commencing statins mean LDL-C was reduced by 37% to 70.8 mg/dL (*P* < 0.01) with a corresponding 24% (*P* < 0.01) reduction in apoB. Mean LDL-C at 12 months (79.3 g/dL) appeared slightly greater than at 3 months, but this was not statistically significant. Triglycerides and CRP did not change significantly at 3 or 12 months, though HDL cholesterol did show significant increase by 12 months – lipid and apolipoprotein data are shown in Table 2.

Compared to baseline, at 3 months there were no significant changes in the proportion of patients taking either angiotensin converting enzyme inhibitors (12/24 versus 8/24: *χ*^2^ = 0.77; *P* = 0.38) or beta adrenoceptor blockers (22/24 versus 18/24: *χ*^2^ = 1.35; *P* = 0.25). By 12 months, the number of patients taking ACE inhibitors had increased significantly compared to baseline (17/24 versus 8/24: *χ*^2^ = 5.3; *P* *<* 0.05), though this would not have influenced the study findings at the early time point of 3 months. Beta blocker usage was not changed at 12 months (19/24 versus 18/24: *χ*^2^ = 0.11; *P* = 0.74). There was no significant change in either blood pressure or heart rate over the course of the study (Table 3).

### 3.2 Atherosclerosis regression

All 24 patients had aortic plaque images of sufficient quality for analysis. Four patients had carotid plaque images at one or more time points of insufficient quality for analysis; therefore they were excluded from statistical analysis. Images (total > 100) from four randomly selected patients were analysed by both observers yielding inter-observer coefficients of variation for plaque index of 4.8% in the aorta, and 2.9% in the carotid. In both the aorta (Fig. 1a) and the carotid arteries (Fig. 2a), there were statistically significant reductions in plaque index after 3 months of statin treatment. Mean aortic plaque index decreased from 0.303 ± 0.024 at baseline to 0.296 ± 0.022 at 3 months (*P* < 0.05 versus baseline) and 0.288 ± 0.024 at 12 months (*P* < 0.01 versus baseline). Plaque regression in the aorta between 3 and 12 months was also significant (*P* < 0.05). In the carotid artery, plaque index fell from 0.446 ± 0.053 at baseline to 0.432 ± 0.046 at 3 months (*P* < 0.05 versus baseline) and 0.416 ± 0.032 at 12 months (*P* < 0.01 versus baseline). Plaque index for the carotid artery between 3 and 12 months did not show significant reduction (*P* = 0.09). The number of patients with early regression in the aorta at 3 months compared to baseline was 18/24, similar to that at 12 months where 20/24 showed regression (*χ*^2^ = 0.126, *P* = 0.72) (Fig. 1c). The number of patients with measurable early regression at 3 months in the carotid was 14/20, which was not significantly different to the 18/20 observed at 12 months (*χ*^2^ = 1.4, *P* = 0.24); see Fig. 2c. Furthermore within patients, the early (3 months) change in plaque index of aorta and carotid arteries showed significant correlation with the final change observed at 12 months (Figs. 1c and 2c).

Mean aortic lumen area was 431 ± 77 mm^2^ at baseline, 434 ± 77 mm^2^ at 3 months (*P* = 1.0 versus baseline) and 442 ± 85 mm^2^ at 12 months (*P* = 0.09 versus baseline). Mean lumen area in the carotid arteries was 44 ± 9 mm^2^ at baseline, 44 ± 8 mm^2^ at 3 months (*P* = 1.0 versus baseline) and 43 ± 8 mm^2^ at 12 months (*P* = 0.55 versus baseline).

### 3.3 Physiological measures

After 3 months of statin treatment, aortic distensibility increased by >20% at each of the three locations along its length. This effect was sustained but did not increase further at 12 months (Table 3). Flow-mediated dilatation of the brachial artery, a measure of endothelial function, also improved after 3 months by >30% (Table 3). Endothelial independent relaxation induced by GTN was not significantly changed after 3 months, but did show a significant increase by 12 months.

### 3.4 Relationship between variables

Within individual patients there was no correlation between MRI quantification of atheroma burden in the aorta and carotid at baseline. Although at a group level, endothelial function and aortic compliance improved and atheroma burden diminished, there was no correlation of these changes within individual patients. Furthermore, there were no significant associations between measures of vascular function or atheroma burden and any of: attained LDL-C; change in LDL-C, HDL-C, apoB, apoA-I, or CRP.

## 4 Discussion

In this study, we have observed that regression of atherosclerosis in response to statin treatment can occur earlier than previously appreciated in both the aorta and carotid arteries. The robustness of this observation is enhanced by the finding that, within individual patients, regression at the early time point of 3 months was closely related to the magnitude and direction of change at 12 months. Patients also showed early and sustained improvement in aortic distensibility and in flow mediated vasodilatation of the brachial artery.

The magnitude of atheroma regression observed in our study after 3 months is consistent with previous longer-term studies of regression in response to statin treatment [6,7,25]. A lesser LDL-C reduction than that achieved in our study was not associated with early plaque regression after 6 months statin treatment [6]. Intensive LDL-C reduction has been reported to achieve greater regression, but this study did not include an early time point [7]. Thus, the early regression observed here is a new finding that accords with the analysis of Nissen et al. [5] in which atheroma regression, assessed by intravascular ultrasound in the coronary arteries, was predicted by attained mean LDL-C < 70 mg/dL. A recent observational study reported overall carotid wall area progression by 2.2% per year [8], although individual cases of regression of up to 7.9% were reported. Importantly, the population in that study differed by the inclusion of older patients with a higher prevalence of hypertension and selected only patients with carotid plaques of ≥50% stenosis on a prior duplex scan.

Mean lumen size of the aorta and carotid appeared to show slight increase by 12 months, this did not reach statistical significance implying that regression was occurring predominantly by reversal of ‘positive’ vessel remodelling as suggested by Corti et al. [6]. There was significant change in plaque index between 3 and 12 months in the aorta, whilst the carotid artery showed a trend that did not reach significance (*P* = 0.09) over the same time period. This could reflect the smaller size of the carotid artery compared to the carotid, making a small change harder to detect. Alternatively the plaques in the aorta and carotid may differ in composition, with certain elements of the plaque likely to be more susceptible to removal than others. For instance, lowering plasma LDL-C could slow the rate of lipoprotein deposition in the arterial wall, allowing reverse cholesterol transport mechanisms to predominate, culminating in net regression [26]. Different effects on individual plaque components is suggested by MRI studies of advanced carotid atherosclerosis in which patients treated with aggressive lipid lowering therapy showed a reduction in size of lipid rich areas [27,28]. This study was not designed to examine plaque composition, though initiation of study to address this question has recently been reported [29]. New developments such as lipid selective contrast agents or higher field strength (3 T) imaging with improved resolution might further establish the degree to which plaque regression involves lipid removal [30,31].

Early clinical benefits of statins have also been ascribed to anti-inflammatory effects [32]. The degree of change in coronary atheroma volume measured using intravascular ultrasound has been related to the magnitude of change in both LDL and CRP [33]. However, in the present study, the early changes in plaque size occurred in the absence of significant change in CRP. Lack of measurable change in CRP probably reflects both the relatively small sample size, and the low baseline CRP level in this stable CAD population, comparable to the post treatment CRP levels in other studies [34]. We also found no relationship between the LDL-C attained and change in plaque size, which may again reflect the sample size and a clustering of LDL-C levels in the lower range precluding observation of a quantitative effect. However, the absence of changes in biochemical parameters despite plaque regression highlights the complementary role of MRI as an imaging biomarker.

Rapid improvements in endothelial vasomotor function have previously been demonstrated in patients with atherosclerosis within weeks [12,14] or even days [13] of starting statin treatment. Thus our finding of increased flow mediated dilatation at 3 months was not surprising. By contrast, timing of statin effects on central arterial stiffness is less established. Improvements in large artery stiffness have been demonstrated after a year of statin treatment [35,36], although a shorter study of 8 weeks pravastatin treatment in patients with familial hypercholesterolemia did not show any improvement [37]. Hypertensive patients without coronary artery disease treated with high dose atorvastatin (mean LDL of <70 mg/dL) did show improvements in large artery stiffness after 3 months [38]. Our finding of rapid improvement in aortic stiffness was of similar magnitude and extends this potentially beneficial effect of statin treatment to a coronary artery disease population. We also observed that GTN mediated (endothelium independent) dilatation of the brachial artery was increased after 12 months statin treatment, as has been previously reported [39,40] and which might reflect downstream statin effects e.g. on smooth muscle cell sensitivity to nitric oxide.

We initially hypothesised that changes in arterial function might predict changes in structural parameters, but in this study we found no correlation on an individual patient basis between changes is structure and function. Endothelial function, aortic stiffness and atheroma burden all represent different aspects and stages of disease. Although at a population level all these parameters may change in a favourable direction in response to treatment, individual patients may show variable response in each according to their stage of disease. As a result, it is likely that measures of vascular structure and function will provide complementary insights into vascular disease [18].

The capability of MRI to perform non-invasive assessment of changes in the arterial wall using relatively small numbers of patients is highlighted by this and other studies [6,22]. Accurate and reproducible assessment of the vessel wall is key to detection of atherosclerosis progression or regression [41]. The rationale for this approach has been illustrated by previous carotid intima-media thickness or coronary IVUS studies [42–44]. An early appreciation of effects on both structure and function, as provided by MRI, could be used to guide selection of novel agents prior to investment in major Phase III trials [45]. MRI is an emerging technique and unlike ultrasound measures of IMT and FMD, has not yet been validated as a means to predict future events in large studies. However, recent data shows correlation of aortic plaque burden by MRI with clinical risk scores [46]. As our findings suggest MRI potentially offers additional value through the assessment of arterial structure in multiple locations and complementary measures of vascular function. Therefore, it seems increasingly likely MRI will play a key role in evaluation of new and existing therapies and may even become part of individual patient risk assessment.

### 4.1 Study limitations

A potential limitation of this study is the absence of a control group. However, given strong evidence of the benefits of early and intensive treatment with statins, a placebo controlled arm or even low intensity statin-treatment arm was not considered ethical. In common with several recent studies [5,6], we therefore conducted a longitudinal study of changes compared to baseline. As a result, it is not possible to exclude the possibility that the changes observed were due to an unknown factor, though in context, this is improbable. These patients were newly diagnosed with coronary artery disease, some of whom were taking vasoactive drugs such as ACE inhibitors and beta adrenoceptor blockers for hypertension prior to study enrolment. However, the increase in use of such medications between study baseline and the 3-month time point was small and not statistically significant and so the vascular changes observed over that period are not likely to be confounded by changes in these other medications. The 2–3% reductions in plaque index observed in this study at 3 months appear relatively modest when compared to decreases in lesion size of greater than 30% observed over a similar time frame in some animal studies. However, the interventions in animal models of atherosclerosis usually involve much more extreme changes in lipid levels than those achievable by statin treatment in humans. Thus, whilst statin treatment to achieve effective LDL-C reduction appears important for plaque regression, additional HDL-C based interventions in order to enhance reverse cholesterol transport may prove even more effective [26]. The patient population studied was overwhelmingly male and Caucasian therefore the findings may not be applicable across all population groups.

## 5 Conclusions

This study shows that in a population of statin naïve, clinically stable but otherwise unselected coronary artery disease patients, cholesterol reduction using statins to mean LDL-C of approximately 70 mg/dL was associated with rapid regression of atheroma at 3 months. Early changes were highly correlated with changes after 12 months. These rapid structural changes were accompanied by early improvements in arterial stiffness and endothelial function that were sustained to 12 months. Use of multi-modal vascular MRI to detect early changes in atheroma and vascular function in small numbers of patients could prove to be an efficient strategy to screen novel anti-atherosclerotic agents.

## Figures and Tables

**Table 1:** Baseline characteristics of patients

Number of subjects (M/F)	24 (20/4)
Age (years)	66.0 ± 8.7
Weight (kg)	80.0 ± 11.6
Body mass index (kg/m^2^)	26.9 ± 3.6
History of diabetes (%)	2 (8%)
History of hypertension (%)	11 (46%)
History of smoking (%)	15 (63%)

Statin treatment	
Simvastatin 40 mg daily (%)	15 (63%)
Simvastatin 10–20 mg daily (%)	5 (21%)
Atorvastatin 10 mg daily (%)	4 (17%)

Concomitant medication	
ACE-inhibitor or angiotensin-II blocker	8 (33%)
Beta-blocker	18 (75%)
Aspirin	20 (83%)

**Table 2:** Lipid measures and C-reactive protein

	Baseline	3 months	12 months
Total-C (mg/dL)	187.9 ± 41.0	147.2 ± 28.9*	154.2 ± 27.2*
LDL-C (mg/dL)	112.7 ± 38.8	70.8 ± 23.1*	79.3 ± 22.7*
HDL-C (mg/dL)	43.9 ± 16.4	47.8 ± 23.3	49.7 ± 16.2*
Apo B (mg/dL)	90.2 ± 25.1	68.5 ± 15.1*	71.1 ± 15.0*
Apo A-I (mg/dL)	133.9 ± 29.2	132.1 ± 28.1	129.4 ± 23.4
Triglycerides (mg/dL)	157.7 [89.5–201.1]	123.1 [85.9–194.0]	117.8 [78.8–158.5]
hsCRP (mg/L)	1.8 [1.2–3.0]	1.7 [0.7–6.8]	0.9 [0.5–4.3]

* *P* < 0.01 vs. baseline.

**Table 3:** Parameters of vascular function

	Baseline	3 months	12 months
Aortic distensibility			
Ascending	1.86 ± 1.23	2.56 ± 2.05*	2.41 ± 1.31*
Proximal descending	2.36 ± 1.24	3.00 ± 1.44*	3.16 ± 1.52*
Distal descending	3.56 ± 2.19	4.34 ± 2.77**	5.40 ± 2.68*

Brachial artery (%*Δ*)			
FMD	8.6 ± 4.6	11.4 ± 5.5*	13.7 ± 5.4*
GTN	31.6 ± 16.5	35.6 ± 17.2	40.6 ± 11.3*

Heart rate (bpm)	57 ± 9	55 ± 10	55 ± 8
Systolic BP (mmHg)	130 ± 17	127 ± 19	124 ± 14
Diastolic BP (mmHg)	77 ± 12	75 ± 11	74 ± 9

* *P* < 0.05 vs. baseline.

** *P* = 0.05 vs. baseline.
